# Acceptability, Usability, and Perceived Quality of Care of a Digital Decision Support System in Tele–Primary Health Care in Sindh, Pakistan: Sequential Explanatory Mixed Methods Pilot Study

**DOI:** 10.2196/81995

**Published:** 2026-06-11

**Authors:** Samrah Jawed, Hasan Nawaz Tahir, Anum Shaikh, Hamza Aziz, Sara Saeed Khurram, Iffat Zafar Aga, Muhammad Muzzamil, Mahek Karim, Abdul Momin Kazi, Shifa Salman Habib

**Affiliations:** 1 Department of Paediatrics and Child Health, Aga Khan University, National Stadium Road, Aga Khan University Hospital, Stadium Road ,P.O. Box 3500 Karachi, Karachi, 74800, Pakistan, 922134864811; 2 Department of Community Medicine, Shaqra University, Dawadmi, Riyadh Region, Kingdom of Saudi Arabia; 3 Department of Community Health Sciences, Aga Khan University, Karachi, Pakistan; 4 Department of Pathology, Ziauddin University, Karachi, Pakistan; 5 Community Innovation Hub, Sehat Kahani, Karachi, Pakistan

**Keywords:** digital decision support systems, telehealth, primary health care, female physicians, technology acceptance, Pakistan

## Abstract

**Background:**

Primary health care (PHC) delivery in Pakistan is constrained by persistent workforce shortages, which are further exacerbated by the attrition of trained female physicians following marriage or childbirth. Telehealth platforms, such as Sehat Kahani, have emerged as one response to this gap, enabling female physicians to provide remote primary care from home. Within this model, a digital decision support system (DDSS) was recently piloted for selected febrile illnesses to strengthen clinical decision-making. However, evidence on how such systems are perceived by female PHC providers in low-income and middle-income country settings remains limited. In particular, there is limited understanding of how perceived usefulness, ease of use, and perceived impact on quality of care shape the early adoption of DDSS within tele-PHC workflows.

**Objective:**

This pilot study aimed to explore the acceptability, perceived ease of use, and perceived quality of care associated with a DDSS among female PHC providers delivering teleconsultations through a large-scale telehealth platform in Sindh, Pakistan.

**Methods:**

An exploratory pilot study using a sequential explanatory mixed methods design was conducted. Quantitative data were collected through an online survey of female health care providers (N=30) across 5 telehealth clinics using the DDSS. The survey assessed experiences related to DDSS utilization, usability, technical facilitation, satisfaction, and perceived diagnostic and treatment accuracy. This was followed by 3 focus group discussions to further examine facilitators and barriers to DDSS use. Survey data were analyzed descriptively, and qualitative data were analyzed thematically. Qualitative findings were used to explain and contextualize quantitative patterns.

**Results:**

Survey findings indicated frequent integration of DDSS into routine teleconsultation practice, with 43.3% (n=13) of providers using the system multiple times per day. DDSS was primarily used alongside clinical judgment (n=16, 53.3%) rather than as a standalone decision-making tool. Half (n=15, 50%) of participants reported confidence in the accuracy of DDSS-supported recommendations, while 46.7% (n=14) reported occasional reassessment of system outputs. Usability perceptions were generally positive, with 50% (n=15) reporting moderate satisfaction and 46.7% (n=14) finding the system easy to navigate. Qualitative findings contextualized these patterns, highlighting that DDSS enhanced decision-making confidence, supported care in unfamiliar clinical domains, and promoted standardized practice, while also revealing concerns about algorithm completeness and workflow burden.

**Conclusions:**

This pilot study suggests that DDSS embedded within telehealth platforms is acceptable to female PHC providers and can support clinical decision-making in resource-constrained PHC settings. By providing early implementation evidence from a real-world tele-PHC setting in a low- or middle-income country, this study contributes context-specific insights to inform iterative refinement and responsible scale-up of DDSS-enabled care models.

## Introduction

Primary health care (PHC) is widely recognized as the foundation for achieving equitable and accessible health care. It is defined as a whole-of-society approach that aims to ensure the highest possible level of health and well-being, along with their equitable distribution, by focusing on people’s needs along the continuum from health promotion and disease prevention to treatment, rehabilitation, and palliative care, as close as feasible to people’s everyday environments [1]. PHC is considered essential for attaining “health for all” and strengthening health systems through accessible, high-quality, people-centered care [1,2]. Despite this central role, many low- or middle-income countries (LMICs), including Pakistan, face persistent challenges in delivering effective PHC due to critical workforce shortages and inequitable health care provider distribution. Prior studies have identified Pakistan as 1 of the 57 countries experiencing a significant imbalance in health care provider availability, with shortages particularly pronounced in rural and underserved areas [3]. These workforce constraints undermine the delivery of accessible and high-quality PHC services, resulting in gaps in service coverage, continuity of care, and quality monitoring. In such contexts, quality, although a core principle of PHC, often remains insufficiently addressed, despite robust evidence demonstrating that poor-quality care contributes to more deaths globally than lack of access to health care [4].

Sindh, the second-largest province in Pakistan by population, exemplifies these challenges. Approximately 46.03% of Sindh’s population resides in rural areas [5], and socioeconomic vulnerability remains high, with an estimated 44.7% of Pakistan’s population living below the World Bank’s lower-middle income country poverty line of US $4.20 per capita per day [6]. Although Sindh has an extensive public-sector health infrastructure, including teaching hospitals, district and subdistrict hospitals, rural health centers, basic health units, and dispensaries, many rural facilities remain under-resourced, understaffed, and underused, contributing to persistent gaps in access to and quality of PHC. In this context, the private sector plays a substantial role in health care delivery, accounting for approximately 60% of health care use in the province [7].

Within Pakistan’s constrained health system, gender-based workforce attrition further exacerbates provider shortages. A significant proportion of trained female physicians withdraw from conventional facility–based practice due to sociocultural norms, safety concerns, and caregiving responsibilities, despite representing a substantial investment in human capital. In this study, female PHC providers refer specifically to medically qualified physicians delivering first-contact, comprehensive primary care through teleconsultations, including clinical assessment, diagnosis, treatment, and referral.

Telehealth has emerged as a pragmatic strategy to address workforce and access challenges in LMICs such as Pakistan, particularly during and following the COVID-19 pandemic. By enabling remote consultations, telehealth has helped bridge gaps in service delivery and continuity of care in health systems constrained by rapid population growth and limited resources [8]. Increased penetration of mobile devices and internet connectivity has further facilitated the integration of mobile health solutions and telemedicine into PHC, extending services to underserved rural and peri-urban populations [9]. In Pakistan, private sector telehealth platforms have played a particularly important role in re-engaging female physicians who are unable to participate in conventional facility–based care. Platforms such as Sehat Kahani employ female physicians to deliver teleconsultations from home-based settings, offering an innovative response to both workforce shortages and gender-based barriers in health care delivery [10].

Building on telehealth platforms, digital health interventions, such as a digital decision support system (DDSS), have the potential to further strengthen PHC delivery. DDSS are computer-based tools that generate patient management recommendations by synthesizing patient data with evidence-based clinical algorithms and health information inputs. These systems support health care providers in diagnostic reasoning, treatment selection, and adherence to clinical guidelines, thereby improving the quality and consistency of care [11]. DDSS are particularly relevant in resource-limited settings, where high patient volumes and limited diagnostic capacity increase the risk of diagnostic errors, posing significant threats to patient safety and health outcomes [12,13].

Evidence from LMICs suggests that DDSS can play a meaningful role in strengthening PHC systems when embedded within routine clinical workflows. In India, the integration of a clinical decision support system into the Comprehensive Primary Health Care–Non-Communicable Diseases program improved the management of hypertension and diabetes by supporting nonspecialist providers, enhancing adherence to treatment protocols, and improving the quality of care in primary health settings [14]. Similarly, in South Africa, the Practical Approach to Care Kit (PACK) represents a well-evaluated clinical decision support intervention specifically designed for PHC settings in LMICs. Developed over nearly 2 decades, PACK provides structured, symptom-based, evidence-informed guidance aligned with local disease burden, national clinical policies, and resource constraints. Evidence from the PACK program demonstrates improvements in guideline adherence, consistency of care, and provider confidence, while functioning not merely as a technical tool but as a cognitive and educational support embedded within broader health system strengthening efforts [15].

Despite rapid advances in digital health technologies, the acceptability, perceived ease of use, and perceived impact on the quality of care among health care providers remain underexplored, particularly in LMIC settings. Moreover, the perspectives of female PHC providers delivering care through telehealth platforms are notably underrepresented in the digital health literature, despite their critical role in addressing workforce shortages in countries such as Pakistan. While prior studies have examined DDSS in primary care, limited empirical evidence exists on how such tools are perceived and used by female physicians delivering tele-PHC, particularly in contexts characterized by gender–based workforce attrition and resource constraints.

Moreover, existing literature has largely focused on system performance, guideline adherence, or clinical outcomes, with comparatively limited attention to provider-level perceptions that shape early implementation and sustained use. Understanding how constructs such as perceived usefulness, ease of use, and perceived impact on care quality operate within gendered tele–primary care environments remains critical for informing responsible digital health scale-up in resource-constrained settings.

This study, therefore, aims to explore the acceptability, perceived ease of use, and perceived quality of care associated with a DDSS among female PHC providers delivering teleconsultations through a large-scale private telehealth platform in Sindh, Pakistan. By focusing on providers actively using DDSS in real-world tele-PHC settings, this study contributes early implementation evidence on technology acceptance among female physicians in an LMIC context and generates contextually grounded insights to inform the design, optimization, and scale-up of digital decision support tools in resource-constrained health systems.

**Figure 1.**
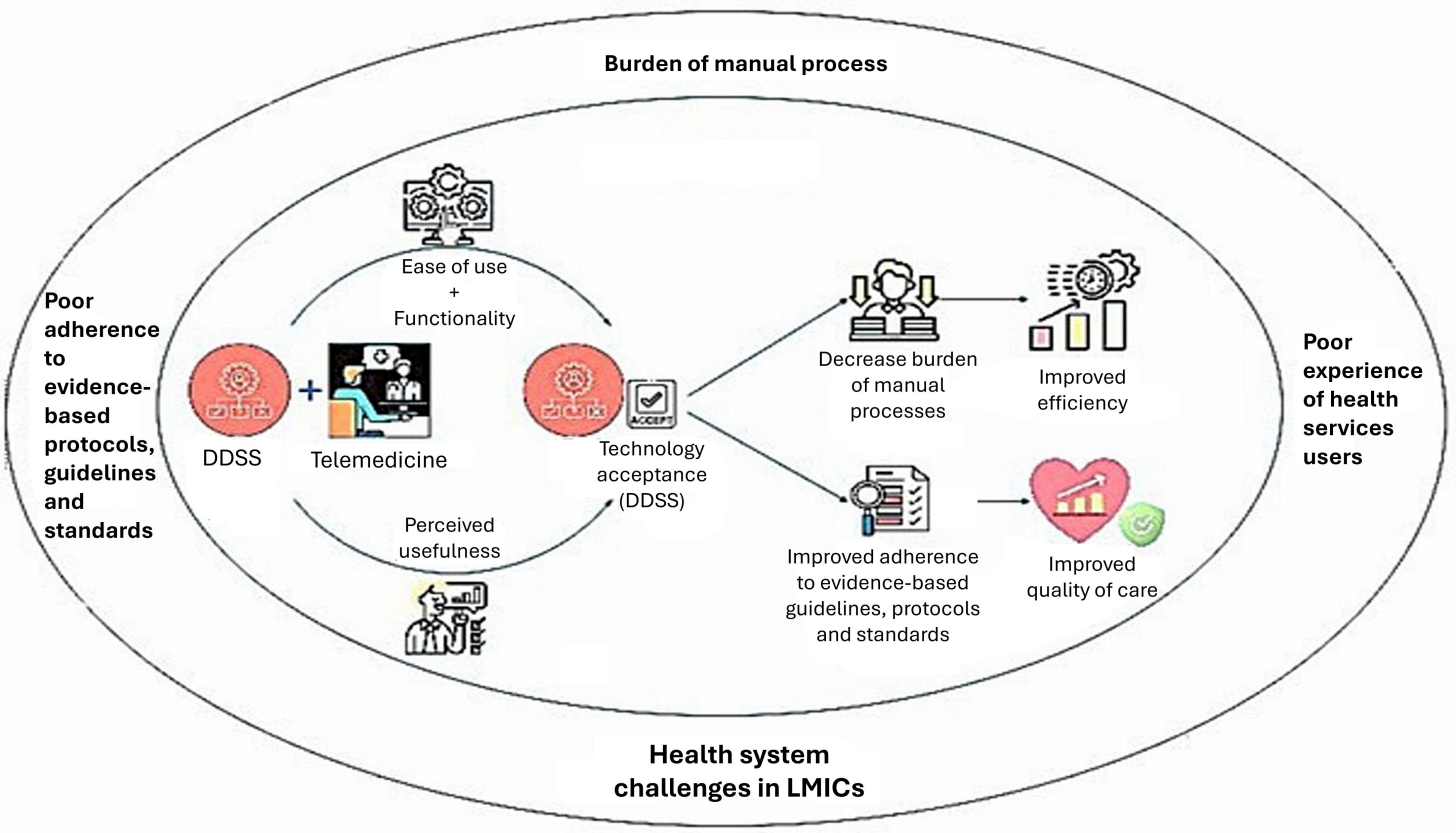
Conceptual framework adapted from the World Health Organization (WHO)’s tool for monitoring and evaluation of digital health interventions and the technology acceptance model [16]. DDSS: digital decision support system; LMIC: low- or middle-income country.

The conceptual framework guiding this study was adapted from the World Health Organization (WHO)’s monitoring and evaluation framework for digital health interventions and the technology acceptance model (TAM), as outlined in the published study protocol (Figure 1) [16]. The WHO framework provides a system-level lens for assessing digital health implementation, including usability, workflow integration, and quality-of-care dimensions. TAM, in contrast, focuses on individual-level determinants of technology adoption, particularly perceived usefulness and perceived ease of use. By integrating these frameworks**,** this study examines both system-level and provider-level dimensions influencing early adoption of DDSS within tele-PHC settings.

## Methods

### Ethical Considerations

Ethical approval for this study was obtained from the ethical review committee at Aga Khan University, Karachi (#2023-8514-25036). All participants provided informed verbal consent prior to participation. Confidentiality and anonymity were strictly maintained. The study was conducted in accordance with the Declaration of Helsinki.

### Study Design

This study was conducted as an exploratory pilot study using a sequential explanatory mixed methods design. The design was selected to first generate descriptive quantitative insights into health care providers’ experiences with a newly piloted DDSS, followed by qualitative exploration to explain and contextualize survey findings. The qualitative phase was intentionally designed to explain unexpected, divergent, or context-dependent quantitative findings and to deepen understanding of provider-level perceptions. This approach was appropriate given the exploratory nature of the pilot and the need to characterize patterns of DDSS use before examining contextual factors influencing provider perceptions. As the DDSS was recently introduced in a limited number of telehealth clinics and covered a narrow range of common febrile conditions, including pneumonia, typhoid, malaria, dengue, and urinary tract infections, a pilot design was considered appropriate to assess feasibility, acceptability, and early user experiences prior to large-scale implementation or hypothesis testing.

### Study Setting

This study was conducted across 5 private telehealth clinics operated by Sehat Kahani, in rural and peri-urban regions of Sindh, Pakistan. At the time of the study, all selected clinics were actively using a DDSS embedded within the telehealth platforms as part of routine teleconsultation workflows for selected febrile illnesses.

Sehat Kahani is a private digital health organization that provides PHC services through teleconsultation models in underserved communities. The platform employs female physicians, including those unable to participate in conventional facility–based practice due to sociocultural or caregiving constraints, to deliver first-contact primary care remotely using digital technology. The research team was not involved in the development or implementation of the DDSS and conducted the evaluation independently to minimize potential response bias.

### Sampling and Participants

A nonprobability purposive sampling strategy was used to recruit participants. Eligible participants were medically qualified female PHC providers affiliated with Sehat Kahani telehealth clinics, where the DDSS had been implemented.

Inclusion criteria included the active provision of teleconsultations using the DDSS and a minimum of 3 months’ experience working on the telehealth platform augmented with the DDSS. Health care providers working in clinics where the DDSS was not implemented or those who did not provide consent were excluded.

The study included health care providers from 5 telehealth clinics, each staffed by approximately 6 providers, resulting in a total survey sample of 30 participants. As the DDSS had been implemented in only 5 clinics at the time of the study, all eligible providers were invited to participate, representing a census of active DDSS users within the selected clinics. All 30 eligible providers completed the survey (response rate: 100%).

All survey participants were subsequently invited to participate in the qualitative component. A total of 3 focus group discussions (FGDs) were conducted, each comprising approximately 10 providers. Participants were grouped according to work shifts (morning, evening, and night shifts) to facilitate scheduling and maximize participation. As all eligible providers were invited and participated in the focus group discussions, the qualitative component aimed to capture the full range of perspectives among active DDSS users within the selected sites rather than to achieve theoretical saturation.

### Data Collection

Data collection was conducted between November 2023 and December 2023. Quantitative data were collected first using a structured online survey administered via Google Forms. The survey assessed health care providers’ experiences with the DDSS. Survey items were adapted from previously published studies evaluating DDSS and technology acceptance constructs [17,18] and were aligned with the study’s conceptual framework [16]. As the instrument was adapted for the tele-PHC context, it was reviewed by subject matter experts in digital health and primary care to ensure content validity and contextual relevance. Given the pilot nature of the study and the small sample size, formal psychometric validation was not undertaken. The survey required approximately 5 to 10 minutes to complete.

Following a preliminary review of the survey findings, qualitative data were collected through FGDs to further explore perceived facilitators and barriers to DDSS use. A semistructured interview guide was developed based on the survey results, focusing on usability, clinical integration, trust, workflow implications, and perceived impact on the quality of care.

FGDs were conducted online in English using Zoom (Zoom Communications, Inc). English was used as the medium of discussion, as it is the routine language of professional communication among the participating physicians. Sessions were audio-recorded with participants’ consent and lasted approximately 45 to 50 minutes each. A total of 2 members of the research team, trained in qualitative methods, facilitated the discussions, while field notes were taken to document contextual information, participant interactions, and salient points arising during the discussions.

### Data Analysis

Quantitative data were analyzed descriptively to summarize health care providers’ experiences with the DDSS. Survey responses were summarized using frequencies and percentages, consistent with the exploratory and pilot nature of the study. No inferential statistical testing was undertaken. Descriptive analyses were conducted using Stata software (version 17; StataCorp).

Qualitative data were analyzed using a thematic analysis approach, guided by the study’s conceptual framework and informed by survey findings, in accordance with the sequential explanatory design.

Audio recordings were transcribed verbatim, and transcripts were imported into NVivo software (version 12; Lumivero) to support data management and coding. An initial deductive coding structure was informed by key areas explored in the survey and conceptual framework, while allowing for the inductive identification of additional themes emerging from the data. A total of 2 members of the research team independently coded the transcripts, followed by iterative comparison and refinement of codes into subthemes and overarching themes. Differences in interpretation were resolved through discussion and consensus to enhance analytical rigor. Integration occurred at the interpretation stage, where qualitative findings were used to explain and contextualize quantitative results in line with the sequential explanatory mixed methods design.

## Results

### Demographic and Professional Profile of Study Participants

All survey respondents (N=30) were female health care providers delivering teleconsultations through the Sehat Kahani platform. Most participants were general practitioners (n=21, 70%), while the remainder were specialists (n=9, 30%). With respect to their highest qualifications, half of the participants held an MBBS degree (n=15, 50%), followed those with by clinical fellowship or membership qualifications (n=9, 30%), master’s degrees (n=3, 10%), and doctoral degrees (n=3, 10%).

In terms of professional experience, most participants reported between 1 and 10 years of practice, with 40% (n=12) having 1 to 5 years of experience and 33.3% (n=10) having 6 to 10 years of experience. Fewer participants reported longer durations of practice, including 11 to 15 years (n=4, 13.3%), 16 to 20 years (n=3, 10%), and more than 20 years (n=1, 3.3%). The primary areas of expertise were general practice (n=19, 63.3%), maternal and child health (n=6, 20%), and infectious diseases (n=3, 10%), with a small proportion reporting expertise in mental health (n=1, 3.3%) and emergency medicine (n=1, 3.3%). Participant characteristics are summarized in Table 1.

**Table 1.** Demographic and professional characteristics of female primary health care providers (N=30) participating in a sequential explanatory mixed methods pilot study of a digital decision support system embedded in a tele–primary health care platform in Sindh, Pakistan, November 2023-December 2023.

Characteristics	Values, n (%)	
Gender		
Female	30 (100)	
Designation		
General practitioner	21 (70)	
Specialist	9 (30)	
Highest level of education		
Bachelor’s degree	15 (50)	
Master’s degree	3 (10)	
Doctorate degree	3 (10)	
Clinical fellowship and membership	9 (30)	
Years of professional experience		
1-5	12 (40)	
6-10	10 (33.3)	
11-15	4 (13.3)	
16-20	3 (10)	
<20	1 (3.3)	
Primary area of expertise		
General practice	19 (63.3)	
Maternal and child health	6 (20)	
Infectious diseases (eg, HIV/AIDS, malaria, and tuberculosis)	3 (10)	
Mental health	1 (3.3)	
Emergency medicine	1 (3.3)	

### Quantitative Survey Findings

Survey findings showed patterns of DDSS use within teleconsultation practice. From a total of 30 participants, 43.3% (n=13) reported using the DDSS multiple times per day, while 40% (n=12) reported using it several times per week. DDSS use was primarily integrated with clinical judgment rather than used in isolation; 53.3% (n=16) of providers reported combining DDSS recommendations with their own clinical expertise, and none relied exclusively on the system for decision-making. The DDSS was most commonly used in complex clinical cases (n=15, 50%), followed by situations involving time constraints (n=10, 33.3%).

Confidence in DDSS-generated recommendations varied among participants. 50% (n=15) of the participants reported being confident in the accuracy and reliability of DDSS-supported diagnoses, while 36.7% (n=11) reported neutral confidence. Nearly half of the respondents (n=14, 46.7%) expressed varying levels of agreement regarding the integration of DDSS into clinical practice. Most participants either strongly agreed (n=13, 43.3%) or agreed (n=12, 40%) that DDSS should be integrated into health care delivery, and 50% (n=15) reported being highly inclined to recommend the system to colleagues. Peer influence appeared supportive but not dominant, with 33.3% (n=10) reporting moderate influence and 46.7% (n=14) indicating that they had received positive feedback from many colleagues.

60% (n=18) of participants described the DDSS as highly accessible within routine workflows, and 76.7% (n=23) reported the presence of specific organizational policies supporting its use.

Technical support was rated as very responsive (n=14, 46.7%) or responsive (n=12, 40%) by most participants, while training support was similarly rated as good (n=13, 43.3%) or excellent (n=12, 40%).

Participants reported differing levels of receptiveness to continuing DDSS use. Most participants reported being moderately (n=18, 60%) or highly receptive (n=7, 23.3%) to ongoing use, despite 46.7% (n=14) reporting minor concerns related to system functionality. Perceived effectiveness was moderate, with 50% (n=15) describing the DDSS as moderately effective in facilitating clinical practice. Half of the respondents (n=15, 50%) also reported significant improvements in adherence to clinical management guidelines following DDSS implementation.

Participants reported varying levels of satisfaction and perceived usability. A total of 56.7% (n=17) of participants reported being moderately satisfied with the DDSS’s impact on patient care, while 50% (n=15) reported moderate satisfaction with the system’s user interface. Nearly half of the respondents (n=14, 46.7%) found the system very easy to navigate, and most rated the DDSS-generated recommendations as either very clear (n=13, 43.3%) or moderately clear (n=12, 40%).

Participants reported both complete and partial inclusion of diagnostic and treatment components within the DDSS. While 50% (n=15) of participants reported that DDSS disease management protocols were completely aligned with established guidelines, many noted only partial inclusion of diagnostic components (n=14, 46.7%) and treatment components (n=15, 50%). Disease management planning and follow-up components (n=15, 50%) and risk factor information (n=15, 50%) were most frequently described as partially included, indicating perceived gaps in clinical comprehensiveness. Detailed distributions of survey responses across domains are presented in Table 2.

**Table 2.** Survey responses on utilization, acceptability, usability, and perceived quality of care related to the digital decision support system (DDSS) among female primary health care providers in Sindh, Pakistan.

Survey item and response category		Values, n (%)	
Domain 1: utilization pattern			
Frequency of using DDSS in teleconsultation			
Multiple times a day		13 (43.3)	
Several times a week		12 (40)	
Once a week or less		4 (13.3)	
Rarely		1 (3.3)	
Decision-making during teleconsultations			
Rely on clinical experience and knowledge only		12 (40)	
Rely on DDSS only		0 (0)	
Combination of DDSS and clinical experience		16 (53.3)	
Collaborative input from others then validate through DDSS		2 (6.7)	
Factors influencing use of DDSS for decision-making			
Complexity of case		15 (50)	
Time constraint		10 (33.3)	
Recommendation from another health care provider		5 (16.7)	
Confidence in accuracy and reliability of diagnosis using DDSS			
Very confident		3 (10)	
Confident		15 (50)	
Neutral		11 (36.7)	
Not confident		1 (3.3)	
Reconsidering decisions after using DDSS			
Rarely		12 (40)	
Occasionally		14 (46.7)	
Frequently		1 (3.3)	
Always		3 (10)	
Domain 2: peer Influence			
Inclination to recommend DDSS to other health care professionals			
Highly inclined		15 (50)	
Moderately inclined		10 (33.3)	
Neutral		2 (6.7)	
Not inclined		3 (10)	
Influence of colleagues on use of DDSS for decision-making			
High influence		7 (23.3)	
Moderate influence		10 (33.3)	
Low influence		5 (16.7)	
No influence		8 (26.7)	
Perceived feedback from colleagues on using DDSS			
Positive feedback from many colleagues		14 (46.7)	
Positive feedback from few colleagues		11 (36.7)	
No feedback from colleagues		5 (16.7)	
Negative feedback from colleagues		0 (0)	
Domain 3: technical facilitation by telehealth platform			
Accessibility of DDSS in teleclinic practice			
Highly accessible		18 (60)	
Moderately accessible		11 (36.7)	
Minimally accessible		1 (3.3)	
Not accessible		0 (0)	
Organizational policies or guidelines to promote use of DDSS			
Specific policies or guidelines for DDSS present		23 (76.7)	
General policies or guidelines (not DDSS-specific) present		5 (16.7)	
Not sure about policies or guidelines for DDSS		1 (3.3)	
No policies or guidelines present		1 (3.3)	
Perceived level of support and training provided for DDSS			
Excellent		12 (40)	
Good		13 (43.3)	
Fair		3 (10)	
Poor		2 (6.7)	
Responsiveness of technical support team in resolving technical issues			
Very responsive		14 (46.7)	
Responsive		12 (40)	
Neutral		4 (13.3)	
Not responsive		0 (0)	
Domain 4: users’ behavioral intention			
Integrating DDSS in health care practice			
Strongly agree		13 (43.3)	
Agree		12 (40)	
Disagree		5 (16.7)	
Strongly disagree		0 (0)	
Receptiveness to continue use of DDSS in clinical practice			
Highly receptive		7 (23.3)	
Moderately receptive		18 (60)	
Minimally receptive		4 (13.3)	
Not receptive		1 (3.3)	
Concerns or reservations about using DDSS in clinical practice			
No concerns or reservations		11 (36.7)	
Minor concerns or reservations		14 (46.7)	
Moderate concerns or reservations		5 (16.7)	
Major concerns or reservations		0 (0)	
Adherence to patient management guidelines due to DDSS			
Significantly improved adherence		15 (50)	
Moderately improved adherence		7 (23.3)	
No change in adherence		8 (26.7)	
Reduced adherence		0 (0)	
Effectiveness of DDSS in facilitating clinical practice			
Very effective		9 (30)	
Moderately effective		15 (50)	
Minimally effective		6 (20)	
Not effective		0 (0)	
Domain 5: user satisfaction			
Satisfaction with impact of DDSS on patient care and other health outcomes			
Very satisfied		6 (20)	
Moderately satisfied		17 (56.7)	
Minimally satisfied		7 (23.3)	
Dissatisfied		0 (0)	
Satisfaction with overall user interface and usability of DDSS			
Very satisfied		6 (20)	
Moderately satisfied		15 (50)	
Minimally satisfied		9 (30)	
Dissatisfied		0 (0)	
Ease of use to manage and navigate technology			
Very easy		14 (46.7)	
Easy		11 (36.7)	
Difficult		5 (16.7)	
Very difficult		0 (0)	
Clarity in the DDSS-generated recommendations			
Very clear		13 (43.3)	
Moderately clear		12 (40)	
Minimally clear		5 (16.7)	
Unclear		0 (0)	
Domain 6: accuracy of diagnosis and treatment			
Alignment of disease management with established international guidelines			
Completely aligned		15 (50)	
Partially aligned		13 (43.3)	
Not aligned		0 (0)	
Not sure		2 (6.7)	
Inclusion of comprehensive diagnostic plan in DDSS			
Completely included		13 (43.3)	
Partially included		14 (46.7)	
Not included		1 (3.3)	
Not sure		2 (6.7)	
Inclusion of comprehensive treatment plan in DDSS			
Completely included		12 (40)	
Partially included		15 (50)	
Not included		0 (0)	
Not sure		3 (10)	
Inclusion of disease management plan (follow-up and lifestyle modification) in DDSS			
Completely included		8 (26.7)	
Partially included		15 (50)	
Not included		6 (20)	
Not sure		1 (3.3)	
Inclusion of information on risk factors for disease in DDSS			
Completely included		9 (30)	
Partially included		17 (56.7)	
Not included		1 (3.3)	
Not sure		3 (10)	

### Qualitative Findings From Focus Group Discussions

Qualitative findings from the FGDs expanded on and contextualized the survey results, providing deeper insight into health care providers’ experiences with the DDSS. Two overarching categories emerged: facilitators to DDSS use and barriers to DDSS use, each comprising multiple themes. These themes broadly aligned with survey domains related to perceived usefulness, ease of use, confidence, and clinical comprehensiveness, thereby supporting the integration of quantitative and qualitative findings.

#### Facilitators to Use DDSS

##### Theme 1: General Acceptability and Perceived Usefulness of DDSS

Participants consistently described the DDSS as a useful and acceptable component of teleconsultation practice. The structured, algorithm-based approach was perceived as supportive in guiding clinical reasoning, particularly by prompting consideration of differential diagnoses and reinforcing standardized care pathways. Providers emphasized that the system complemented, rather than replaced, their clinical judgment:

*It’s a very good tool; by following it, you can easily reach the diagnosis. At least, it gives an idea of what to think about and what other differentials could be there. If we use it alongside our clinical knowledge, works very well.*

[Participant 2]

Several participants highlighted that the use of standardized protocols promotes consistency in decision-making and alignment with clinical guidelines. One participant stated, “It is very effective; in this way, uniformity is being maintained, and protocols and guidelines are being followed” (Participant 18).

##### Theme 2: Enhanced Confidence in Clinical Decision-Making

Participants reported that the DDSS enhanced their confidence during clinical decision-making, particularly in situations involving uncertainty. The availability of structured algorithms and embedded recommendations was perceived as providing reassurance that clinical decisions were aligned with established guidelines. One participant stated, “It provides an edge and confidence in the way you are managing your patient” (Participant 11).

This confidence was reinforced by the perception that DDSS-supported diagnoses were generally accurate and consistent with guidelines for the conditions covered by the system. One participant stated, “The added algorithms, like pneumonia and malaria, are very helpful, and they are accurate as per the guidelines” (Participant 28).

##### Theme 3: High Perceived Ease of Use and Workflow Integration

Ease of use emerged as a prominent facilitator of DDSS adoption. Participants described the system interface as intuitive and user-friendly, noting that features such as drop-down menus, predefined clinical questions, and checklist-based formats reduced cognitive burden and supported efficient consultations. One participant stated, “There’s a checklist, so approaching it, reading it, and then examining the patient becomes very easy” (Participant 21).

The simplicity of language and clear structure were viewed as enabling use across various levels of technical proficiency. One participant stated, “The options are very simple in language, so it can be easily filled by anyone” (Participant 26).

##### Theme 4: Support for Managing Unfamiliar Clinical Domains

Participants emphasized that the DDSS was particularly valuable when managing conditions outside their primary areas of expertise. The system was perceived as extending diagnostic reach across specialties and supporting decision-making in less familiar clinical scenarios:

*The most significant benefit I foresee from this tool is for other specialties where we don’t have much experience. Through this tool, we can integrate the diagnosis of other specialties.*

[Participant 2]

Several participants suggested that the DDSS could serve as a useful learning resource for less experienced providers, including early-career clinicians. One participant stated, “It especially helps us when we are confused, and I believe it’s a great tool for interns as well” (Participant 13).

### Barriers to Use of DDSS

#### Theme 1: Incomplete Algorithms and Limited Disease Coverage

A major barrier identified by participants was the limited scope of conditions covered by the DDSS. Providers noted that the system primarily focused on febrile illnesses and lacked algorithms for several common and contextually relevant conditions, thereby reducing its applicability in routine practice:

*Currently, it only covers fever, and even within the fever category, there are many details and aspects missing. Hopefully, with time, it will improve and become more comprehensive.*

[Participant 27]

Participants emphasized the need for broader disease coverage and more comprehensive diagnostic pathways to enhance the system’s clinical use. One participant stated, “There are many diagnoses not on the list that should be included as well” (Participant 6).

#### Theme 2: Increased Documentation Burden

Participants reported that DDSS use increased documentation workload due to duplication across multiple forms. This was perceived as time-consuming and as detracting from patient care during teleconsultations. One participant stated, “We have to fill out the 3 forms, and there is repetition of information” (Participant 1).

Providers suggested that integrating DDSS documentation into a single streamlined form could improve efficiency and usability. One participant stated, “Having a comprehensive single form for that would be helpful” (Participant 1).

#### Theme 3: Mistrust Related to Diagnostic Comprehensiveness

Participants expressed reservations regarding reliance on DDSS-generated diagnoses in complex cases, particularly when symptoms overlapped across infectious diseases. The absence of alternative diagnostic considerations in some algorithms led providers to question the completeness of the system’s recommendations. One participant stated, “Sometimes the clinical picture mimics each other making it difficult to differentiate...we get the final diagnosis without consideration for other diseases” (Participant 2).

As a result, providers emphasized the importance of retaining clinical oversight and using DDSS as a supportive tool rather than as a definitive decision-maker. One participant stated, “The end decision is still in the hands of the doctor” (Participant 4).

## Discussion

### Principal Findings and Comparison With Prior Work

The findings of this study highlight both the potential and the practical challenges of integrating DDSS into PHC delivery through telehealth platforms in LMICs such as Pakistan. To our knowledge, this is the first study from Pakistan to explore female PHC providers’ perceptions of the acceptability, ease of use, and perceived quality of care associated with a DDSS embedded within a telehealth platform. This study contributes early implementation evidence from a low-resource tele–primary care setting and provides context-specific insights into facilitators and barriers influencing provider-level adoption of digital decision support tools.

As previously noted, clinical decision-making is increasingly characterized by the ability to access and apply the best available evidence in real time [19]. DDSS represents a practical example of this shift by providing health care professionals with rapid access to structured diagnostic guidance within routine workflows. Overall, DDSS was positively received, particularly in relation to usability, technical facilitation, and enhanced confidence in clinical decision-making. These findings are consistent with prior research demonstrating that DDSS can support diagnostic accuracy and promote adherence to clinical guidelines in resource-constrained settings [9-20]. The system was largely perceived as a supportive tool that complemented clinical judgment while reinforcing standardized care pathways.

These results align with the TAM, which identifies perceived usefulness and perceived ease of use as key determinants of behavioral intention to adopt digital systems [21]. High reported usability, workflow integration, and willingness to continue use suggest that provider acceptance was influenced by both functional utility and system design within the telehealth environment.

Participants’ experiences are also consistent with evidence from both high-income and LMIC settings indicating that algorithm-based decision support tools can improve workflow efficiency and reduce cognitive burden through structured prompts [19,22]. Increased provider confidence observed in this study mirrors broader trends in the adoption of evidence-informed clinical tools, particularly in settings where access to specialist expertise is limited [11,12]. In such contexts, DDSS may help reduce diagnostic uncertainty and potential errors [23].

However, important limitations were identified. Narrow disease coverage, incomplete algorithms, and documentation burden contributed to occasional mistrust in DDSS-generated recommendations. These findings echo earlier research emphasizing that the clinical utility and adoption of DDSS are highly dependent on the breadth, contextual relevance, and completeness of embedded algorithms [23]. The qualitative findings provided important nuance to the quantitative results. While 50% of participants reported confidence in DDSS-supported diagnoses, FGDs indicated that this confidence depended on algorithm completeness and contextual relevance. Similarly, survey-reported minor reservations were explained by the duplication of documentation and limited diagnostic breadth. These findings underscore that adoption and sustained use depend on iterative system refinement, expanded disease coverage, and improved workflow integration. Interoperability with existing health information systems may further reduce fragmentation and documentation burden.

This study also contributes to the limited literature examining digital health adoption through a gender-specific lens. In Pakistan, many trained female physicians disengage from conventional clinical practice due to sociocultural and caregiving constraints [10]. Embedding DDSS within telehealth platforms may enhance professional confidence and autonomy while supporting the retention of this workforce segment.

From a theoretical perspective, this study extends technology adoption research by situating DDSS acceptance within a gendered telehealth workforce model in an LMIC context. Practically, the findings emphasize the importance of algorithm expansion, workflow optimization, and context-sensitive system refinement to sustain provider trust and long-term adoption.

Overall, contextually adapted DDSS integration within telehealth platforms may strengthen tele-PHC delivery in Pakistan by supporting nonspecialist providers and improving access to essential services in underserved settings.

### Strengths and Limitations

This study has several strengths. The sequential explanatory mixed methods design enabled the integrated interpretation of quantitative and qualitative findings. The focus on female health care providers foregrounds an under-represented segment of the workforce central to telehealth delivery in Pakistan. Conducting the study within active telehealth clinics ensured that the findings reflect real-world implementation.

Limitations should also be acknowledged. The exclusive inclusion of female health care providers and restriction to Sindh province may limit generalizability. The pilot scope of the DDSS, covering selected febrile illnesses, constrains its applicability to other disease areas. Additionally, the cross-sectional design captures perceptions at an early implementation stage. Longitudinal research is needed to examine evolving usage patterns and impacts on care quality over time.

### Conclusions

This exploratory pilot study provides early implementation insights into the acceptability, perceived ease of use, and perceived quality of care associated with a DDSS embedded within a telehealth platform in Sindh, Pakistan. The findings suggest that the DDSS can serve as a supportive clinical tool for female PHC providers by enhancing decision-making confidence, reinforcing standardized care pathways, and facilitating teleconsultation workflows when integrated into routine practice.

However, implementation challenges—including limited disease coverage, incomplete algorithms, and increased documentation burden—affected provider trust and the perception of the clinical comprehensiveness of the system. These findings highlight the importance of ongoing system refinement, expanded clinical scope, and improved workflow integration to ensure responsiveness to provider needs and local care contexts.

Although the results are context-specific, they provide implementation-relevant evidence to inform the design and potential scale-up of DDSS-enabled telehealth models in resource-constrained settings. Future longitudinal and multisite research is essential to assess sustained adoption, broader clinical applicability, and impacts on care quality and health system performance. Overall, this study contributes early empirical evidence on digital decision support in PHC and underscores its potential role in strengthening telehealth delivery and supporting underrepresented segments of the health care workforce.

## Abbreviations

DDSS: digital decision support system
FGD: focus group discussion
LMIC: low- or middle-income country
PACK: Practical Approach to Care Kit
PHC: primary health care
TAM: technology acceptance model
WHO: World Health Organization
